# Observrtions on the Salivary Glands in the Four Classes of Vertebrated Animals

**Published:** 1804-12-01

**Authors:** M. Duvernoy


### Observrtions on the Salivary Glands in the four Classes of vertebrated Animals, by M. Duvernoy.

The author has made his observations on a great number of animals from each of the four classes, the results of which are as follow:

1. The mammifera are the only animals provided with glands belonging to the order of the conglomeratæ.

2. These glands are, however, not to be met with in the cetacea, as has already been noticed by Mr. Cuvier, in his memoir on the porpus (Delphinus Phocœna, L.) and the porpoise (Delphinus Delphis, L.)

3. They are proportionably smaller in the amphibious mammifera than in the rest.

4. The glandulæ parotideæ, and sublinguales are wanting, which is never the case with the submaxillares. Thus the myrmecophaga, and the echidna, have submaxillares and sublinguales, the first of which are very considerable, but the same animals have no parotides.

5. The herbivorous animals are possessed of a much more considerable salivary system, than is found in the carnivorous kind, which result, though not new, is confirmed by a great number of observations.

6. In the carnivorous and gnawing animals, *(rongeurs)* it often happens, that the proportions of the glandulæ maxillares increase, while that of the parotides decrease, which is sometimes so much the case, that the latter are considerably smaller than the former, as in didelphis virginiana, in the genus vespertilio, canis, phoca, mussylvaticus, phascoloma, &c. and it may be also observed in the raca, raca, and the rabbit. These observations seem to indicate a relation between the manner in which the aliments are submitted to the action of the teeth, and the place where the salivary glands issue their liquor. Hence it appears, that in the carnivorous and gnawing kind of animals, in the first of which the canine and incisive teeth, and in the latter the incisives only, perform the most essential part of mastication; the saliva is, on the whole, conducted in a much greater quantity towards the teeth, than in animals where those teeth have not so essential a function. In the tatu, however, and the sloth, the maxillary glands are likewise much larger than the parotides.

7. In the carnivorous tribe the salivary glands are in general much redder, more composed of lobes, and of a firmer texture.

8. The ductus stenonianus passes not always through the masseter on its way to the buccinator. In the tatu, the pachyderma, the ruminant animals, and the solipeda, it follows the inferior margin of the former, forming there an arch, the convex part of which is turned downwards.

9. It is often the case, that the sublinguales have one canal only, opening at the side of that of the maxillares, which has been observed in the simiæ, several carnivorous animals, and the ruminants. In the solipeda, they have several small canals; in the hog, there are two pairs of them, the anterior of which is large and flat with several small excretory canals; whereas the posterior is long, narrow, and provided with only one canal.

10. The molares generally form a considerable prolongated mass, which is situated opposite the superior teeth of the same name, or also near the inferior molar teeth, as in the cat.

11. The buccales and labiales are in general inconsiderable.

12. Some animals have, besides these glands common to the human species, another gland, which, in some, appears to be but a continuation of the molares; it arises beneath the zygomatic arch, behind the os submaxillare, and opens at the extremity of the superior margo alveolaris with sundry small excretory canals, which takes place in oxen, the sheep, and the horse. In the dog, it is separated from the molares, forming a distinct mass, that has only one excretory canal, opening in the same region. This is the same gland which has been described by Nuck in the dog. J. G. Duvernoy has observed it in the serval. The author, however, could not trace it in the cat.

 13. In

13. In birds, the glands, analogous to the salivary glands of the mammifera, correspond with respect to their situation only to the sublinguales of the latter. They are a collection of small round hollow grains, containing a thick viscous humour, which is conducted to the basis of the palate by numerous small orifices; they are considerable in the gallinacea, but much smaller in the birds of prey, and very minute in water birds. There are two pairs of them in the gallinaceous tribe and in the genus certhia; whereas, in the rest, only one pair is to be found.

14. In reptiles, they have frequently the same granulary structure as is observed in lizards, and the tupinamis, both belonging to the genus lacerta. In these animals they are placed immediately under the skin, along the exterior surface of the branches of the maxilla inferior. In the same manner it is found in serpents which have a thin, long, smooth, and forked tongue. In the amphisbœna, they have also a granulary structure, but they are placed under the tongue between the musculi genioglossi and geniohyoidei. In the greatest part of the other reptiles, the tongue itself seems to consist of a glandulous texture, and its function to be analogous to that of the preceding glands. This gland is very obvious in the ranæ and the lacertæ, the gecko, iquana, draco, chamæleon, and stinci; in all which animals the surface of the tongue is covered with hollow papillæ, in which the salivary humour is separated. In the Greek tortoise, a quantity of small canals are seen on the tongue, which are combined at their basis, and the body thus formed is perforated with many small openings; the tongue of several ranæ is likewise formed of a gelatinous substance.

15. In fishes there is no gland analogous to the salivary glands of the other classes. The rajæ, however, and the squali are provided with glandulous grains, which are situated immediately under the membrane of the palate, opposite the cartilago transversalis, which answers the os hyoideum, and the great muscular depressor maxillæ inferioris. They seem to pass their humour at the basis of the palate, though this could not be observed on applying a strong pressure. The other fishes offer nothing similar, but they have, like the former, two glandulous strata, more or less thick at the origin of the œsophagus, betwixt the interior and the muscular membrane. It is doubtful, whether they ought to be referred to the salivary glands, or whether they be more analogous to the palatic glands of of birds, or the amygdalæ of the mammifera, which seem to have been placed nearly in the same spot, in order to envelope the aliments on their passage into the œsophagus.

